# Crude Extract and Phenol-Rich Fractions from *Vernonia amygdalina* Leaves Ameliorates Streptozotocin-Induced Type 1 Diabetes in Rats by Mitigating Hepatic Injury, Dyslipidemia, and Production of Oxido-Inflammatory Markers

**DOI:** 10.3390/jox16020053

**Published:** 2026-03-20

**Authors:** Olawale Razaq Ajuwon, Damilola Rebecca Oladejo, Akinwunmi Oluwaseun Adeoye, John Adeolu Falode, Basiru Olaitan Ajiboye, Foluso Oluwagbemiga Osunsanmi, Babatunji Emmanuel Oyinloye

**Affiliations:** 1 Redox Biology Research Laboratory, Department of Biochemistry, Federal University Oye-Ekiti, Oye-Ekiti P.M.B. 373, Ekiti State, Nigeria; anikedamilola@gmail.com; 2 Biomembrane, Phytomedicine, and Drug Development Unit, Department of Biochemistry, Federal University Oye-Ekiti, Oye-Ekiti P.M.B. 373, Ekiti State, Nigeria; akinwunmi.adeoye@fuoye.edu.ng; 3 Biomembrane, Molecular Pharmacology, and Toxicology Laboratory, Department of Biochemistry, Federal University Oye-Ekiti, Oye-Ekiti P.M.B. 373, Ekiti State, Nigeria; john.falode@fuoye.edu.ng; 4 Phytomedicine and Molecular Toxicology Research Laboratory, Department of Biochemistry, Federal University Oye-Ekiti, Oye-Ekiti P.M.B. 373, Ekiti State, Nigeria; basiru.ajiboye@fuoye.edu.ng; 5 Biotechnology and Structural Biology (BSB) Group, Department of Biochemistry and Microbiology, University of Zululand, KwaDlangezwa 3886, South Africa; alafin21@yahoo.com (F.O.O.); babatunjioe@abuad.edu.ng (B.E.O.); 6 Institute for Drug Research and Development, Bogoro Research Centre, Afe Babalola University, Ado-Ekiti P.M.B. 5454, Ekiti State, Nigeria; 7 Phytomedicine, Biochemical Toxicology and Biotechnology Research Laboratories, Department of Biochemistry, College of Sciences, Afe Babalola University, Ado-Ekiti P.M.B. 5454, Ekiti State, Nigeria

**Keywords:** *Vernonia amygdalina*, dyslipidemia, oxidative stress, inflammation

## Abstract

Diabetes mellitus (DM) is a major disorder contributing to human mortality and morbidity globally. The use of medicinal plants in the management of diabetes is gaining global popularity due to their accessibility and cost-effectiveness. In this study, we evaluated the ameliorative potential of *Vernonia amygdalina* leaves crude extract (CE), free phenol (FP), and bound phenol (BP) fractions (50 mg/kg body weight) in a rat model of streptozotocin (STZ)-induced type 1 diabetes (T1DM). The effects of these treatments for 28 days on glucose, insulin, glycated hemoglobin, hepatic injury indices, and lipid profile were assessed in the serum. Furthermore, redox biomarkers (liver) and inflammatory mediators (serum and liver) were analyzed. Our results indicated that CE, FP, and BP fractions of *Vernonia amygdalina* inhibited the deleterious effects of T1DM by attenuating hyperglycaemia, insulin deficiency, hepatic injury, and dyslipidemia. Also, CE, FP, and BP fractions differentially improved antioxidant enzymes activity and reduced oxidative and inflammatory markers production. Specifically, CE showed superior effects compared with FP, BP, and metformin across multiple biomarkers, including glycated hemoglobin, α-amylase, α-glucosidase, hepatic glycogen, total cholesterol, LDL-cholesterol, protein carbonyl, SOD, IL-1β, and IL-10. The antidiabetic effects produced by CE, FP, and BP fractions of *Vernonia amygdalina* may be ascribed to the presence of different bioactive phytochemicals as revealed by HPLC analysis. Overall, our data would suggest a potential therapeutic role for *Vernonia amygdalina* leaves extracts in addressing hepatic complications due to T1DM.

## 1. Introduction

Diabetes mellitus (DM) comprises a number of metabolic disorders that is prevalent globally and responsible for significant health expenditure, morbidity, and death. Data from the International Diabetes Federation revealed that, as of 2024, about 589 million adults (20–79 years) are living with diabetes, resulting in an estimated 3.4 million deaths globally [1]. Because of the frightening increase in the prevalence of diabetes, the same report has now projected that the incidence of diabetes will rise to an estimated 853 million people globally by 2050. With a diabetes age-adjusted comparative prevalence rate of 3.6%, and about 2.99 million adults (20–79 years) living with diabetes, Nigeria ranked as the country with the highest incidence of diabetes in sub-Saharan Africa [1]. Therefore, diabetes in Nigeria can be said to have risen to an epidemic proportion. This geometric rise has been adduced to changing lifestyle and behavioral risk factors such as a lack of exercise and sedentary lifestyle, consumption of alcohol, and cigarette smoking [2]. Persistent hyperglycemia is the hallmark of diabetes mellitus, and this may arise as a result of dysfunctional insulin secretion due to autoimmune destruction of β-cells of the pancreas (type 1 DM), or an impaired insulin action leading to insulin resistance (type 2 DM).

Dyslipidemia is the pivotal risk factor for the cardiovascular disorders linked with either the type 1 or type 2 diabetic state. Dyslipidemia arising from DM is characterized by increased fasting and postprandial triglycerides (TG) associated with reduced high-density lipoprotein cholesterol (HDL-C) and elevated or normal low-density lipoprotein-cholesterol (LDL-C), coupled with predominant small dense LDL [3,4]. Factors such as increased mobilization of fatty acid from adipose tissue, effect of insulin on apoprotein formation in the liver, and increased cholesterol biosynthesis have been reported to be the likely drivers of the abnormal increase in serum lipids observed in the diabetic state [5]. Due to hyperglycemia-induced dyslipidemia, DM patients are at greater risk of metabolic disorders such as obesity, hypertension, and cardiovascular disease such as stroke, coronary heart disease, and congestive heart failure [4], which are all leading causes of morbidity and death in DM patients.

Whether in T1DM or T2DM, it has been well-documented that hyperglycemia-induced reactive oxygen species (ROS) generation, and subsequent oxidative stress, is a pivotal upstream occurrence that mediates development and progression of DM. The increased ROS generation that occurs in DM may impact lipids, proteins, and nucleic acids directly, causing structural and functional changes to these macromolecules. For example, ROS-induced oxidative stress has been reported to trigger apoptotic β-cell death, leading to a reduction in β-cell mass and subsequent dysfunction [6]. Other evidence has linked ROS over-generation to the initiation of insulin resistance [7] and the inhibition of insulin secretion from β-cells [8]. Furthermore, a direct association between ROS generation, oxidative stress, and DM comes from the observation that the levels of oxidative stress biomarkers such as malondialdehyde, protein carbonyls, nitrotyrosine, and 8-hydroxy-2-deoxyguanosine are elevated in rodent models as well as in diabetic patients [9]. Equally observed is the fact that the elevation in oxidative damage biomarkers is associated with a reduction in the concentration of non-enzymatic antioxidants, including reduced glutathione (GSH) and vitamins C and E, as well as a decrease in the activities of enzymatic antioxidants [2,10]. A strong association has been reported between oxidative stress and inflammation in the onset and progression of diabetes. Hyperglycemia-induced generation of ROS leads to oxidative stress, which in turn elevates the levels of pro-inflammatory markers such as tumor necrosis factor-alpha (TNF-α), interleukin-1β (IL-1β), and interleukin-6 (IL-6), while reducing anti-inflammatory markers like interleukin-10 (IL-10) [9,11]. Additionally, elevated production of TNF-α has been linked to insulin resistance associated with obesity, a known risk factor for diabetes [9,12]. Moreover, increasing evidence has suggested that the persistent activation of pro-inflammatory pathways in insulin-targeted cells contributes to obesity, insulin resistance, and related metabolic syndromes, including diabetes [13]. Considering that both oxidative stress and chronic inflammation are key processes involved in the pathogenesis of diabetes mellitus, it is hypothesized that herbal plants with known antioxidants and anti-inflammatory properties may offer new therapeutic approaches to the management of DM.

The use of herbal plants in the management and treatment of different disease conditions in low- and medium-income countries around the world is well reported. The medicinal usefulness of these plants has been associated with the abundance of various bioactive phytochemicals (including phenolic acids, alkaloids, sesquiterpenes, flavonoids, tannins, and saponins) with proven benefit in the management and treatment of different disorders. An example of such a plant is *Vernonia amygdalina*, known as Ewuro across the Yoruba-speaking region of Nigeria. *V. amygdalina* (Family: Asteraceae) is a shrubby tropical plant that is endemic to several regions of Africa but is grown as far and wide as Asia [14,15]. *V. amygdalina*, on the account of the bitter taste of the leaves, is referred to as a bitter leaf; however, when macerated and washed, the leaf has been used as a very popular vegetable across all regions in Nigeria. Several parts of *V. amygdalina*, including the leaves, stems, roots, flowers, and seeds have been reported to be rich sources of bioactive secondary metabolite such as flavonoids, tannins, sesquiterpenes, saponins, and alkaloids [14,16,17]. Evidence has shown that the crude extract obtained from the leaves, stems, and roots of *V. amygdalina* showed a wide spectrum of pharmacological effects including antioxidant, bactericidal, antidiabetic, anticancer, anti-inflammatory, and anti-helminthic effects, among others [14,18,19]. However, studies exploring the comparative ability of *V. amygdalina* leaves crude extract and phenolic fractions to inhibit the type 1 diabetes process are limited. Furthermore, the mechanisms through which these phenolic fractions may mediate the potential antidiabetic effect have not been previously studied. This study therefore investigates the antidiabetic, antioxidant, and anti-inflammatory capabilities of the free and bound phenolic fractions obtained from the methanolic crude extract of *V. amygdalina* leaves, using a streptozotocin (STZ)-induced type 1 diabetic rat model.

## 2. Materials and Methods

### 2.1. Chemicals and Reagents

Streptozotocin (STZ), metformin (MET), methanol, acetone, ethyl acetate, sodium hydroxide, concentrated HCl, H_2_O_2_, 5,5′-dithiobis-2-nitrobenzoic acid (DTNB), and glutathione (reduced and oxidized) were purchased from Sigma-Aldrich Chemicals, Steinheim, Germany. Formaldehyde (histological grade), malondialdehyde bis (diethyl acetal) (MDA), and phosphate buffer saline (PBS) were all obtained from Merck Millipore, Darmstadt, Germany. All other chemicals, reagents, and assay kits used were of standard analytical grade and were obtained from reputable manufacturers.

### 2.2. Plant Material Collection and Identification

Matured *V. amygdalina* leaves were collected from Oye-Ekiti, Ekiti State, Nigeria. The identification and authentication of the leaves was carried out by a taxonomist, Dr. F. O. Egbedo of the Department of Plant Biology, University of Ilorin, Nigeria, and a specimen voucher was deposited in the Department’s herbarium (Voucher number: UIH/001/2023).

### 2.3. Extraction of Crude Extract of Vernonia amygdalina Leaves

After thorough washing, *V. amygdalina* leaves were air-dried under the shade at a temperature of 23 ± 2 °C for two weeks. The air-dried leaves were then crushed and pulverized into powder using a Silver Crest electric blender (SC 1589; Guangdong, China). The pulverized leaves (10%, *w*/*v*) were then steeped in a methanol–water mixture (4:1 ratio) for 72 h at room temperature with constant stirring. After 72 h, the macerated plant material was filtered using a Whatman filter paper. The obtained filtrate was concentrated in a rotary evaporator at 40 °C under vacuum to obtain the crude extract (CE) as previously reported by Falode et al. [20].

### 2.4. Extraction of Free Phenol Fraction of Vernonia amygdalina Leaves

Soluble free phenol fraction of *V. amygdalina* leaf was extracted from the crude extract following the method of Chu et al. [21]. Exactly 40 g of CE was extracted in 200 mL of 80% acetone for 10 h at room temperature. The mixture was filtered under vacuum and the residue was kept aside for the extraction of bound phenolic fraction. This was followed by evaporation of about 90% of the filtrate in a rotary evaporator under vacuum at 45 °C to obtain the free phenol (FP) fraction.

### 2.5. Extraction of Bound Phenol Fraction of Vernonia amygdalina Leaves

Bound phenols (BP) fraction present in *V. amygdalina* leaves were extracted from the residue resulting from FP fraction following the method described by Chu et al. [21]. After draining the residue, it was directly hydrolyzed with 20 mL of 4 M NaOH for 60 min at room temperature with constant agitation. The resulting mixture was acidified to pH 2 using concentrated HCl and subjected to six extractions steps with 200 mL of ethyl acetate. The ethyl acetate fractions were combined and evaporated to dryness under vacuum at 45 °C.

### 2.6. High-Performance Liquid Chromatography Analysis of Vernonia amygdalina Extracts

High-performance liquid chromatography (HPLC) analysis of crude extract, free phenol, and bound phenolic fractions of *V. amygdalina* was carried out on a Shimadzu (Nexera XS) HPLC system. The HPLC system consisted of an autosampler (SIL-40C XS), a photodiode array detector (SPD-M30A), a solvent delivery pump (LC-40D XS), and a degassing unit (DGU-405). Separation was carried out at 28 ± 2 °C using a μBondapak C18 (150 mm × 3.9 mm × 10 μm) column serving as stationary phase. The mobile phases consisted of (A) acetonitrile and water acidified with 0.01% formic acid (B). Gradient elution was performed as previously reported by Erukainure et al. [22]. The flow rate was set at 2 mL/min, injection volume at 5 μL, and acquisition of chromatogram set at 254 nm. The concentration of different bioactive compounds in the CE, FP, and BP fractions of *Vernonia amygdalina* were quantified by comparing retention area and/or peak area with standards.

### 2.7. Animals

A total of 54 male Wistar rats (mean weight = 200.78 ± 12.99 g) were housed in the experimental animal house of the Department of Biochemistry, Federal University Oye-Ekiti, Nigeria. The animals were allowed to acclimatize for 10 days under standard conditions of temperature (24 ± 3 °C), 12 h light/dark cycle, and relative humidity (64 ± 3%). During the experiment, animals were allowed free access to standard rat pellets (Ladokun Feeds, Ibadan, Nigeria) and tap water ad libitum. The experiment was conducted following the guidelines outlined in the Guide for the Care and Use of Laboratory Animals (National Institute of Health Publication No. 80-23, revised 1978). Ethical approval for the study was obtained from the Faculty of Science Research Ethics Committee (Certificate number: FUOYEFSC 201122—REC2025/032) of the Federal University Oye-Ekiti, Ekiti State, Nigeria.

### 2.8. Induction of Type 1 Diabetes

After acclimatization for 10 days, overnight fasted rats were checked for their blood glucose level. Type 1 diabetes was then induced in the rats by the intraperitoneal (i.p.) injection of streptozotocin (STZ, 50 mg/kg body weight) dissolved in citrate buffer (0.1 M, pH 4.5) [20]. Seventy-two hours later, rats with fasting blood glucose levels of 250 mg/dL and above (Accu Chek glucometer), were selected as diabetic rats.

### 2.9. Experimental Design

After the confirmation of the onset of diabetes, rats were distributed at random into nine groups, each containing six rats as follows:

Group 1: Control rats.
Group 2: Diabetic untreated rats (50 mg/kg body weight STZ, i.p.) (DM).
Group 3: Diabetic rats + crude extract of *V. amygdalina* (50 mg/kg body weight orally) (DM + CE).
Group 4: Diabetic rats + free phenol fraction of *V. amygdalina* (50 mg/kg body weight orally) (DM + FP).
Group 5: Diabetic rats + bound phenol fraction of *V. amygdalina* (50 mg/kg body weight orally) (DM + BP).
Group 6: Diabetic rats + metformin (200 mg/kg body weight orally) (DM + MET).
Group 7: Crude extract (50 mg/kg body weight orally) only administered rats (CE).
Group 8: Free phenol fraction (50 mg/kg body weight orally) only administered rats (FP).
Group 9: Bound phenol fraction (50 mg/kg body weight orally) only administered rats (BP).

The administered dose (50 mg/kg body weight) of CE, FP, and BP fractions used in the study was based on a previous study by Asante et al. [23], which showed that ethanolic leaf extract of *V. amygdalina* exhibited a pronounced anti-hyperglycemic effect at a dose ranging from 10 to 300 mg/kg body weight in rats. Treatment with CE, FP, and BP fractions of *V. amygdalina* lasted for a period of 28 days with body weight and fasting blood glucose measured routinely. On the 29th day, overnight fasted rats were euthanized with sodium pentobarbital (0.15 mL/100 g body weight) and blood samples collected to obtain serum. The liver from each rat was excised and residual blood removed by rinsing in cold phosphate buffer saline (PBS, 10 mM, pH 7.4) twice. After being blotted to dry, the liver was weighed and divided into two portions. One portion was formalin-fixed for histopathological analysis, and the other portion was immediately homogenized in cold 100 mM potassium phosphate buffer (10%, *w*/*v*, pH 7.4), and the resulting homogenate was centrifuged two times at 10,000× *g* for 12 min at 4 °C. The supernatant obtained was stored at −20 °C for the analysis of hepatic glycogen, oxidative stress indices, and inflammatory markers. The protein concentration of liver homogenate was estimated following the method of Lowry et al. [24].

### 2.10. Biochemical Analysis

#### 2.10.1. Determination of Glucose, Insulin, and Glycated Hemoglobin

Serum glucose (catalog No.: E-BC-K234-S) and insulin (catalog No.: E-EL-R2466) were assayed using commercial kits purchased from Elabscience Biotechnology Company (Wuhan, China) in accordance with instructions of the manufacturer. Glycated hemoglobin (HbA1c; catalog No.: MBS2033689) was determined with rat-specific ELISA kit obtained from MyBioSource Incorporated (San Diego, CA, USA) following the instructions from the manufacturer.

#### 2.10.2. β-Cell Function, Insulin Resistance, and Insulin Sensitivity Indicator Assessment

Homeostasis model assessment of β-cell function (HOMA-β) and homeostasis model assessment of insulin resistance (HOMA-IR) were calculated from fasting insulin and fasting glucose, as reported by Mohammed et al. [25]. Quantitative insulin sensitivity check index (QUICKI) was evaluated, as previously reported by Sasidharan et al. [26]. All the indicators were calculated as shown in Equations (1)–(3).

(1) $$HOMA-\beta =\frac{\left(20\times fasting\;insulin\right)}{\left(fasting\;glucose-3.5\right)}$$

(2) $$HOMA-IR=\frac{\left(fasting\;glucose\times fasting\;insulin\right)}{22.5}$$

(3) $$QUICKI=\frac{1}{\left(log\;insulin+log\;glucose\right)}$$

#### 2.10.3. Determination of Glucose, Lipase, α-Amylase, and α-Glucosidase

The concentration of glycogen in the liver was determined following the protocol outlined by Musabayane et al. [27]. In summary, the liver sample was homogenized in ice-cold 30% potassium hydroxide (1:10 *w*/*v*). The homogenate was subjected to boiling for 30 min at 100 °C, followed by cooling in ice saturated with sodium sulfate. Subsequently, glycogen was precipitated using ethanol, collected as a pellet, and then reconstituted in distilled water. Anthrone reagent (0.2 g anthrone dissolved in 100 mL of concentrated sulfuric acid) was added and the glycogen content determined by measuring absorbance at 620 nm.

Hepatic lipase activity in the serum was assayed using commercial kit (catalog No.: A067-1-2) purchased from Nanjing Jiancheng Institute of Bioengineering (Nanjing, China), in accordance with manufacturer’s instruction.

Serum α-amylase (catalog No.: MBS8243219) and α-glucosidase (MBS8243263) activity were assessed with commercially available kits obtained from MyBioSource Incorporated (San Diego, CA, USA) following instructions of the manufacturer.

#### 2.10.4. Determination of Serum Lipid Profile

Serum triglyceride (catalog No.: A110-1-1), total cholesterol (catalog No.: A111-1-1), high-density lipoprotein cholesterol (HDL-C; catalog No.: A112-1-1) and low-density lipoprotein cholesterol (LDL-C; catalog No.: A113-1-1) were all evaluated with commercial kits obtained from Nanjing Jiancheng Institute of Bioengineering (Nanjing, China). Atherogenic coefficient (AC) and Castelli risk index I (CRI-I) was calculated as shown in Equations (4) and (5), as previously reported by Adedokun et al. [28].

(4) $$AC=\frac{\left(TC-HDL-C\right)}{HDL-C}$$

(5) $$CRI-I=\frac{TC}{HDL-C}$$

#### 2.10.5. Determination of Serum Markers of Liver Function

Activity of alanine aminotransferase (ALT; catalog No.: AL3801), aspartate aminotransferase (AST; catalog No.: AS8306), alkaline phosphatase (ALP; catalog No.: AP8302), and γ-glutamyl transferase (GGT; catalog No.: GT3817) in the serum were all determined using an RX Daytona Automated Clinical Chemistry Analyzer with kits from Randox Laboratory Limited (Dublin, UK) following instructions from the manufacturer.

#### 2.10.6. Determination of Redox Status and Inflammatory Response Markers

Malondialdehyde, a lipid peroxidation index, was determined in liver homogenate according to the method of Beuge and Aust [29]. The extent of protein oxidation was assessed following a previous method reported by Levine et al. [30]. Activity of catalase (CAT) and superoxide dismutase (SOD), as well as the concentration of reduced glutathione (GSH) in the liver homogenate were assayed according to methods of Claiborne [31], Misra and Fridovich [32], and Boyne and Ellman [33] respectively.

Commercial ELISA kits specific for rats were obtained from Elabscience Biotechnology Company (Wuhan, China) and used for the determination of c-reactive protein (CRP; catalog No.: E-EL-R0506) in the serum, as well as hepatic TNF-α (catalog No.: E-EL-R2856), IL-1β (catalog No.: E-EL-R0012), and IL-10 (catalog No.: E-EL-R0016) following the instructions of the manufacturer.

#### 2.10.7. Histopathological Analysis

Histopathological analysis was carried out at the Department of Anatomy and Histology, Afe Babalola University, Ado-Ekiti, Nigeria, following a previous method described by Drury et al. [34]. In brief, the liver sample was paraffin-embedded, sectioned appropriately (4 μm), and stained with hematoxylin and eosin (H&E). A pathologist who was unaware of the study protocol examined and interpreted results from the stained tissue sections.

### 2.11. Statistical Analysis

Data in this study were presented as mean ± SEM. One-way analysis of variance (ANOVA) was used to analyze group mean difference, followed by Tukey post hoc test. Data that were not normally distributed were tested for group differences using the Kruskal–Wallis Test, a non-parametric analog to the one-way ANOVA. GraphPad prism version 5.0 was used for all analysis and graphing, and statistical significance was set at *p* < 0.001, *p* < 0.01, and *p* < 0.05.

## 3. Results

### 3.1. Quantification of Phenolic Compounds in Vernonia amygdalina by HPLC-UV

The results of reverse phase HPLC analyses carried out to quantify the phenolic compounds in the crude extract (CE), free phenol (FP), and bound phenol (BP) fractions of *Vernonia amygdalina* are shown in Table 1 and Figures S1–S3. The results showed the presence of vernomygdin, vernodalin, vernonioside, garanal, vernodalol, myrtenol, luteolin, and benzophenone (not present in crude extract) in the CE, FP, and BP fractions of *V. amygdalina*. It was also observed that luteolin was the bioactive compound that had the highest concentration across the three fractions.

### 3.2. Body Weight of Diabetic Rats Administered Vernonia amygdalina Leaf Extract and Its Phenol-Rich Fractions

The body weight change, liver weight, and relative weight of rats across all experimental groups are shown in Table 2. A significant reduction (*p* < 0.001) in body weight, liver weight, and relative liver weight was observed in untreated DM rats compared with the normal control. Treatment with CE, FP, and BP fractions of *V. amygdalina* resulted in significant improvements (*p* < 0.001) in the body weight, liver weight, and relative liver weight of the DM rats. Body weight changes, liver weight, and relative liver weight of normal rats administered with CE, FP, and BP fractions of *V. amygdalina* were similar to those of normal control rats

### 3.3. Vernonia amygdalina Leaf Extract and Phenol-Rich Fractions Ameliorate Hyperglycemia and Improve Carbohydrate Metabolizing Enzymes in STZ-Induced Diabetic Rats

**Figure 1:**
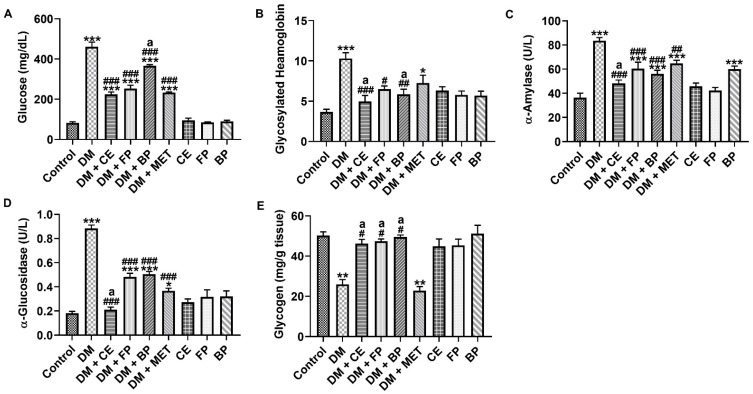
Plasma (**A**) glucose, (**B**) glycosylated hemoglobin, (**C**) α-amylase, (**D**) α-glucosidase, and (**E**) hepatic glycogen levels in STZ-induced diabetic rats after oral administration of crude extract, free phenol, and bound phenol fractions of *V. amygdalina*. Data are expressed as mean ± SEM (*n* = 6). * *p* < 0.05, ** *p* < 0.01, and *** *p* < 0.001 as significant difference versus control. ^#^ *p* < 0.05, ^##^ *p* < 0.01, and ^###^ *p* < 0.001 as significant difference versus DM. ^a^ *p* < 0.05 as significant difference versus DM + MET. BP (bound phenol), CE (crude extract), DM (diabetes), FP (free phenol), MET (metformin).

**Figure 2:**
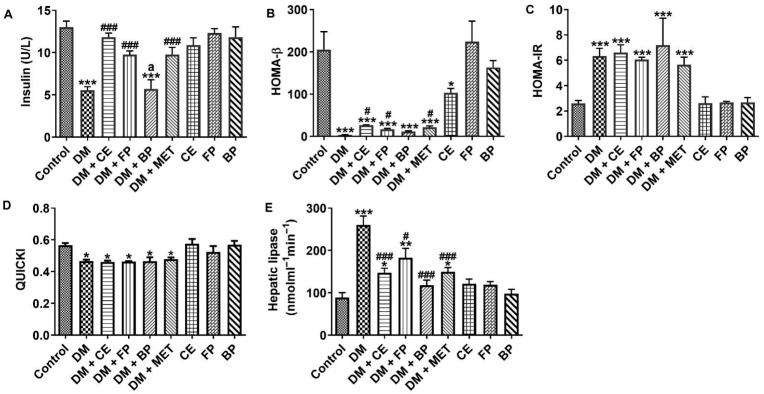
Plasma insulin sensitivity markers showing (**A**) insulin level, (**B**) HOMA-β, (**C**) HOMA-IR, (**D**) QUICKI, and (**E**) hepatic lipase activity in STZ-induced diabetic rats after oral administration of crude extract, free phenol, and bound phenol fractions of *V. amygdalina*. Data are expressed as mean ± SEM (*n* = 6). * *p* < 0.05, ** *p* < 0.01, and *** *p* < 0.001 as significant difference versus control. ^#^ *p* < 0.05 and ^###^ *p* < 0.001 as significant difference versus DM. ^a^ *p* < 0.05 as significant difference versus DM + MET. BP (bound phenol), CE (crude extract), DM (diabetes), FP (free phenol), MET (metformin), HOMA-IR (homeostasis model assessment for insulin resistance), HOMA-β (homeostasis model assessment for β-cell function), QUICKI (quantitative insulin sensitivity check index).

We assessed the anti-hyperglycemic ability of *V. amygdalina* leaf crude extract and its polyphenol-rich fractions by determining the level of glucose and the activities of α-amylase and α-glucosidase in the sera, the level of glycosylated hemoglobin in the whole blood, as well as the glycogen concentration in the livers of rats across all experimental groups. Glucose, glycosylated hemoglobin, α-amylase, and α-glucosidase were all significantly elevated (*p* < 0.001) as a result of STZ induction (Figure 1A, Figure 1B, Figure 1C, and Figure 1D, respectively). Contrastingly, the hepatic glycogen level was significantly depressed in STZ-induced diabetic rats (Figure 1E). The CE, FP, and BP fractions of *V. amygdalina* showed anti-hyperglycemic effects by significantly decreasing the levels of glucose (51.2%, 45.1%, and 25.9% respectively) and glycosylated hemoglobin (51.8%, 36.8%, and 43% respectively), as well as the activities of α-amylase (42%, 27.5%, and 32.7%) and α-glucosidase (71.2%, 45.3%, and 43.6% respectively) in treated diabetic rats compared with untreated diabetic rats (Figure 1A–D). Similarly, CE, FP and BP fractions of *V. amygdalina*, when administered to STZ-induced rats, attenuates the depletion in liver glycogen by increasing its level by 79.4%, 83%, and 91.1%, respectively (Figure 2E). Notably, the anti-hyperglycemic effects shown by CE were significantly higher than the anti-hyperglycemic effects of metformin (standard drug), while both FP and BP fractions demonstrated anti-hyperglycemic actions similar to what was obtained with metformin.

### 3.4. Vernonia amygdalina Leaf Extract and Phenol-Rich Fractions Improves β-Cell Function and Insulin Sensitivity in STZ-Induced Diabetic Rats

Figure 2 shows the effect of *V. amygdalina* leaf extract and its polyphenol-rich fraction on level of insulin, indices of β-cell function, insulin sensitivity, and activity of hepatic lipase in STZ-induced diabetic rats. Serum insulin and HOMA-β levels were significantly decreased (*p* < 0.001) while HOMA-IR levels were significantly elevated (*p* < 0.001) in untreated diabetic rats compared with control rats. Administration of CE and FP fractions to diabetic rats led to significant increases in serum insulin (*p* < 0.001) and HOMA-β (*p* < 0.05), similar to what was obtained in diabetic rats treated with the standard drug, metformin (Figure 2A,B). The observed elevation in HOMA-IR was not significantly affected when CE, FP, BP fractions, and metformin were administered to diabetic rats (Figure 2C). In Figure 2D, the quantitative insulin sensitivity check index (QUICKI) was significantly decreased in diabetic rats, however none of the *V. amygdalina* treatments or metformin had any significant effect on it. Hepatic lipase activity was significantly increased (*p* < 0.001) in untreated diabetic rats compared with control rats (260.0 ± 21.28 versus 88.75 ± 11.55). When CE, FP, BP fractions, and metformin were administered to the diabetic rats, hepatic lipase activity was reduced by 43.35%, 29.69%, 54.61%, and 42.58%, respectively (Figure 2E).

### 3.5. Vernonia amygdalina Leaf Extract and Phenol-Rich Fractions Attenuates Dyslipidemia in STZ-Induced Diabetic Rats

**Figure 3:**
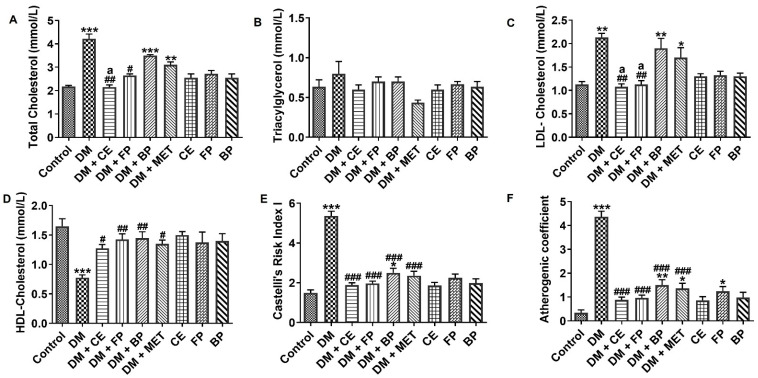
Plasma levels of (**A**) total cholesterol, (**B**) triacylglycerol, (**C**) LDL-cholesterol, (**D**) HDL-cholesterol, (**E**) Castelli’s risk index I, and (**F**) atherogenic coefficient in STZ-induced diabetic rats after oral administration of crude extract, free phenol, and bound phenol fractions of *V. amygdalina*. Data are expressed as mean ± SEM (*n* = 6). * *p* < 0.05, ** *p* < 0.01, and *** *p* < 0.001 as significant difference versus control. ^#^ *p* < 0.05, ^##^ *p* < 0.01, and ^###^ *p* < 0.001 as significant difference versus DM. ^a^ *p* < 0.05 as significant difference versus DM + MET. BP (bound phenol), CE (crude extract), DM (diabetes), FP (free phenol), MET (metformin).

The effects of *V. amygdalina* leaf extract and its phenol-rich fractions on the lipid profile of diabetic rats are shown in Figure 3. In untreated diabetic rats, there was a significant elevation in the plasma levels of TC (*p* < 0.001) and LDL-C (*p* < 0.01), while the level of HDL-C was significantly reduced (*p* < 0.001) (Figure 3A, Figure 3C, and Figure 3D respectively). Similarly in untreated diabetic rats, significant elevation (*p* < 0.001) was observed in the value of calculated Castelli risk index I (CRI-I) and atherogenic coefficient (AC) compared with control rats (Figure 3E,F). Only CE and FP fractions of *V. amygdalina* leaves were able to decrease TC and LDL-C levels when administered to diabetic rats. However, diabetes-induced changes in HDL-C, AC, and CRI-I were all significantly attenuated (*p* < 0.001) by the administration of CE, FP, and BP fractions, as well as MET. Overall, the lipid profile improvement abilities shown by CE and FP fractions were either better or on par with the effect shown by the standard drug, MET.

### 3.6. Vernonia amygdalina Leaf Extract and Phenol-Rich Fractions Ameliorate Diabetes-Induced Liver Dysfunction in Rats

**Figure 4:**
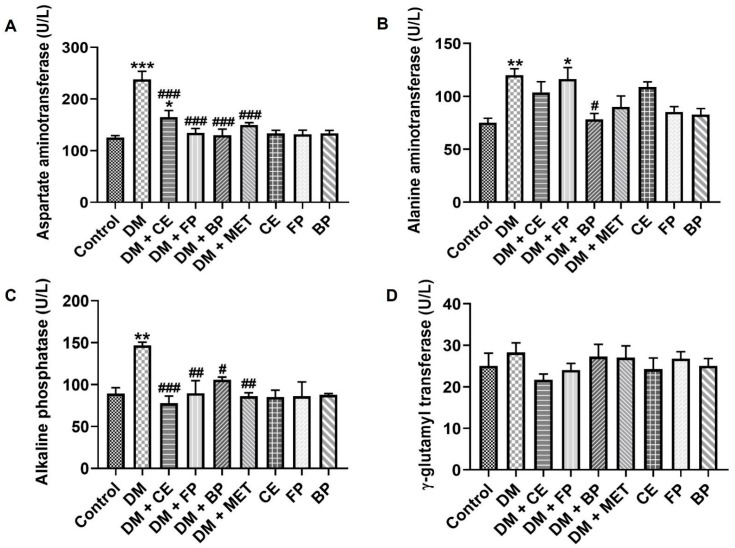
Plasma level of hepatic function markers of STZ-induced diabetic rats after oral administration of crude extract, free phenol, and bound phenol fractions of *V. amygdalina*. (**A**) aspartate aminotransferase, (**B**) alanine aminotransferase, (**C**) alkaline phosphatase, and (**D**) γ-glutamyl transferase. Data are expressed as mean ± SEM (*n* = 6). * *p* < 0.05, ** *p* < 0.01, and *** *p* < 0.001 as significant difference versus control. ^#^ *p* < 0.05, ^##^ *p* < 0.01, and ^###^ *p* < 0.001 as significant difference versus DM. BP (bound phenol fraction), CE (crude extract), DM (diabetes), FP (free phenol fraction), MET (metformin).

Plasma aminotransferase (AST), alanine aminotransferase (ALT), alkaline phosphatase (ALP), and γ-glutamyltransferase (γ-GT) activities were determined to evaluate the protective effect of *V. amygdalina* and its polyphenolic-rich fractions on diabetes-induced liver dysfunction, the results of which are presented in Figure 4. The injection of STZ caused a significant elevation (*p* < 0.001) in the activity of AST (Figure 4A), ALT (Figure 4B), and ALP (Figure 4C) in the plasma of diabetic rats when compared with control rats. Treatment of diabetic rats with crude extract (CE), free phenol (FP), and bound phenol (BP) fractions of *V. amygdalina* attenuated liver dysfunction by significantly decreasing (*p* < 0.05) the activity of AST and ALP when compared with the diabetic untreated rats (Figure 4A,C). Only the BP fraction was able to reduce plasma ALT activity significantly in the diabetic treated rats versus untreated diabetic rats (Figure 4B). Administration of CE, FP, and BP of *V. amygdalina* to rats did not induce any negative effects on AST, ALT, and ALP activities relative to the control rats. Plasma activity of γ-GT did not differ across all experimental groups, as the values were statistically similar (Figure 4D).

### 3.7. Vernonia amygdalina Leaf Extract and Phenol-Rich Fractions Improves Histological Alterations of the Liver in STZ-Induced Diabetic Rats

**Figure 5:**
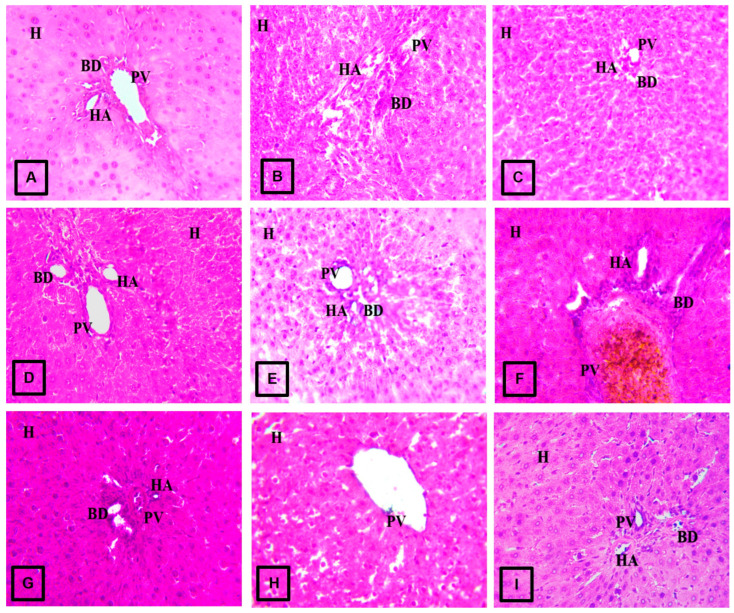
Representative photomicrograph of hematoxylin and eosin (H&E; mag × 800) section of the liver in (**A**) control rats showing normal architecture of hepatocytes with clear portal veins; (**B**) diabetic rats revealing histoarchitecture of the liver showing vacuolar degeneration and disrupted portal areas with hyperplastic walls; (**C**) diabetic rats treated with CE fraction of *V. amygdalina* showing mild degeneration of liver cells and shrunken portal areas; (**D**) diabetic rats treated with FP fraction of *V. amygdalina* showing mild degeneration of the liver cells with clear portal veins; (**E**) diabetic rats treated with BP fraction of *V. amygdalina* showing vacuolar degeneration of liver cells and shrunken portal areas; (**F**) diabetic rats treated with metformin showing hepatocytes with hyperplastic walls of the portal areas and congested portal veins; and (**G**–**I**) normal rats administered CE, FP, and BP fractions of *V. amygdalina* showing normal architecture of liver cells with shrunken portal vein areas. “H, hepatocyte; PV, portal vein; BD, bile duct; HA, hepatic artery”. CE, crude extract; FP, free phenol; BP, bound phenol.

Histological analysis by examining haematoxylin and eosin (H&E)-stained sections of liver tissue corroborated the results from liver function markers. Rats in the control group showed normal architecture of hepatocytes with clear portal veins (Figure 5A). Similarly, H&E sections from normal rats administered CE, FP, and BP fractions of *V. amygdalina* showed normal architecture of the liver cells but with shrunken portal vein areas (Figure 5G, Figure 5H, and Figure 5I, respectively). The stained liver sections of untreated diabetic rats revealed hepatocytes characterized by vacuolar degeneration and disrupted portal areas with hyperplastic walls (Figure 5B). The administration of CE and FP fractions of *V. amygdalina* to diabetic rats significantly improved histological alteration caused by STZ, as their stained liver section revealed mild degeneration of hepatocytes with shrunken portal areas (Figure 5C,D). The administration of BP fraction of *V. amygdalina* to diabetic rats did not improve histological alteration, as the stained sections of their liver still showed gross vacuolar degeneration of hepatocytes with shrunken portal vein areas (Figure 5E).

### 3.8. Vernonia amygdalina Leaf Extract and Phenol-Rich Fractions Improves Hepatic Oxidants and Antioxidants Status in STZ-Induced Diabetic Rats

**Figure 6:**
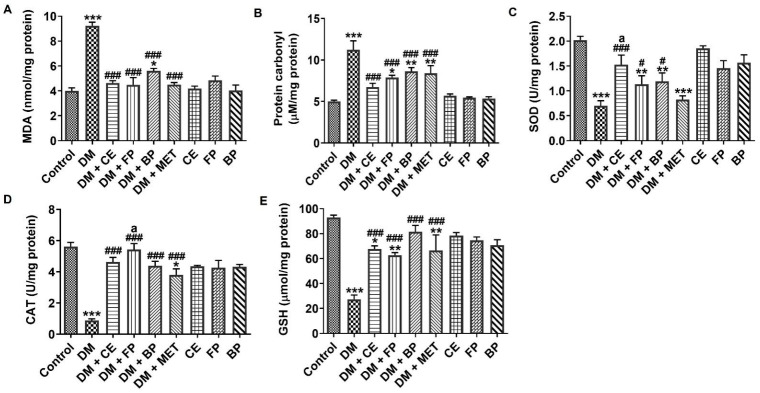
Hepatic oxidant and antioxidant markers of STZ-induced diabetic rats after oral administration of crude extract, free phenol, and bound phenol fractions of *V. amygdalina*. (**A**) Malondialdehyde, (**B**) protein carbonyl, (**C**) SOD, (**D**) CAT, and (**E**) GSH. Data are expressed as mean ± SEM (*n* = 6). * *p* < 0.05, ** *p* < 0.01, and *** *p* < 0.001 as significant difference versus control. ^#^ *p* < 0.05, and ^###^ *p* < 0.001 as significant difference versus DM. ^a^ *p* < 0.05 as significant difference versus DM + MET. BP (bound phenol), CE (crude extract), DM (diabetes), FP (free phenol), MET (metformin).

The status of oxidant and antioxidant markers in the liver of diabetic rats after oral administration of CE, FP, and BP fractions is shown in Figure 6. The injection of STZ resulted in a significant elevation of malondialdehyde (MDA) and protein carbonyls (PCO) level, with a reduction in the activities of SOD, CAT, and GSH levels (Figure 6A–E). The CE, FP, and BP fractions of *V. amygdalina* showed antioxidant effects by significantly inhibiting (*p* < 0.001) the production MDA (Figure 6A) and PCO (Figure 6B), as well as significantly improving (*p* < 0.05) the activity of antioxidant enzymes SOD (Figure 6C) and CAT (Figure 6D), and the level of non-enzymatic antioxidant, GSH (Figure 6E), in the livers of diabetic rats. Overall, the antioxidant effects shown by CE, FP, and BP fractions of *V. amygdalina* were similar to (MDA, PCO, CAT, and GSH) or higher than (SOD) the antioxidant effect shown by the standard drug, MET.

### 3.9. Vernonia amygdalina Leaf Extract and Phenol-Rich Fractions Mitigates Serum and Hepatic Inflammation in STZ-Induced Diabetic Rats

**Figure 7:**
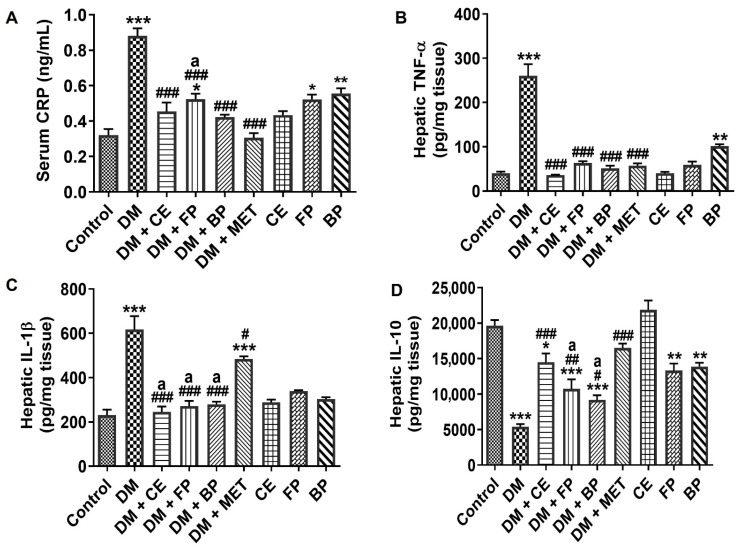
Inflammatory response markers of STZ-induced diabetic rats after oral administration of crude extract, free phenol, and bound phenol fractions of *V. amygdalina*. (**A**) Serum CRP, (**B**) TNF-α, (**C**) IL-1β, and (**D**) IL-10. Data are expressed as mean ± SEM (*n* = 6). * *p* < 0.05, ** *p* < 0.01, and *** *p* < 0.001 as significant difference versus control. ^#^ *p* < 0.05, ^##^ *p* < 0.01, and ^###^ *p* < 0.001 as significant difference versus DM. ^a^ *p* < 0.05 as significant difference versus DM + MET. BP (bound phenol), CE (crude extract), DM (diabetes), FP (free phenol), MET (metformin), and CRP (c-reactive protein).

We assessed the anti-inflammatory effects of CE, FP, and BP fractions of *V. amygdalina* leaves in STZ-induced diabetic rats by measuring the level of CRP in the serum, and levels of TNF-α, IL-1β, and IL-10 in the liver. There was a significant elevation (*p* < 0.001) in the levels of CRP, TNF-α, and IL-1β followed by a significant reduction (*p* < 0.001) in the level of IL-10 in diabetic rats compared with control rats (Figure 7A–D). The administration of CE, FP, and BP fractions to diabetic rats resulted in a significant decrease in the production of CRP (*p* < 0.001) in the serum, as well as TNF-α (*p* < 0.001) and IL-1β (*p* < 0.001) in the liver. Contrastingly, the administration of CE, FP, and BP fractions to diabetic rats led to a significant improvement in IL-10 production in the liver (Figure 7D). The CRP, TNF-α, and IL-1β lowering effect produced by CE, FP, and BP fractions compared favorably with the effect shown by the standard drug, MET. However, while FP and BP fractions improved the production of IL-10, only CE produced an effect similar to what was obtained with MET.

## 4. Discussion

Morbidity and mortality from diabetes is increasing geometrically worldwide despite substantial progress in the understanding of its pathogenesis and improvements in treatment modalities. Therefore, in this study, we employed an in vivo approach to evaluate the antidiabetic, antioxidant, and anti-inflammatory effects of CE, FP, and BP fractions obtained from the leaves of *V. amygdalina* using an STZ-induced rodent model. The pharmacological effects exhibited by natural products, including medicinal plants have been attributed to the presence of bioactive phytochemicals including flavonoids, phenolic acids, tannins, stilbenes, lignans, and others. In our study, HPLC analysis of *V. amygdalina* leaves revealed that luteolin was the most abundant phytochemical in the CE, FP, and BP fractions. Other bioactive compounds identified in these fractions included vernodalin, vernomygdin, garanal, myrtenol, vernodalol, and vernonioside. Our results further showed that the bound phenolic fraction had the highest concentration of the phytochemicals. Previous studies have reported the presence of these compounds, which have been known to possess many health benefits including anticancer, antimicrobial, antioxidant, antimalarial, and anti-inflammatory effects in crude extracts of *V. amygdalina* [16,17]. However, this was the first time that these compounds will have been identified in free and bound phenolic fractions obtained from *V. amygdalina* leaves.

Severe reductions in body weight are one of the most common features and complications of diabetes in human patients as well as animal models. We observed a significant reduction in body weight, liver weight, and relative liver weight in untreated diabetic rats compared with control rats, similar to what has been previously reported [2,35,36]. It has been suggested that DM is a tissue-wasting disorder, and that the decrease in body and organ weight observed in diabetic animals can be attributed to dysfunctional metabolism. This has been reported to arise from a reduction in glucose metabolism and a breakdown of fats and structural proteins, coupled with a reduction in the protein content in muscle tissue due to proteolysis [37,38]. Treatment of diabetic rats with CE, FP, and BP fractions of *V. amygdalina* resulted in a significant improvement in body weight, liver weight, and relative liver weight of the rats. Previously, Atangwho et al. [39] reported that an ethanolic extract of *V. amygdalina* leaves remarkably improved the body weight of STZ-induced diabetic rats, as was observed in this study. This ability to improve the body and liver weights of the diabetic rats may be ascribed to the contributions of various phytochemicals present in the CE, FP, and BP fractions of *V. amygdalina*. Luteolin, the most abundant compound characterized in our extracts, has previously been reported to improve the body and organ weights of diabetic rats by enhancing protein synthesis due to adequate glycemic control [40].

Streptozotocin (STZ) is one of the most used toxicants for the induction of both type 1 and type 2 DM in rodents. STZ can induce a selective necrotic destruction of pancreatic β-cells mediated by alkylation of DNA, accompanied by over-production of ROS and nitric acid (NO), resulting in a state of sustained hyperglycemia [41,42]. Glycation of several proteins that can play important structural and functional roles in the cell is a reported consequence of the sustained hyperglycemia that characterizes the diabetic state [43]. Glycosylated hemoglobin (HbA1c) is a protein that reflects the average glucose level in the blood over a prolonged time (usually 2–3 months) and has been regularly used as a biomarker of glycemic control. Its elevation in the blood can give an indication of a poorly controlled glycemic response, which might increase the risks of micro- and macrovascular complications in the diabetic state [44]. Our results demonstrated that untreated STZ-injected rats presented with marked hyperglycemia, characterized by increased levels of glucose and glycosylated hemoglobin (HbA1c) in the serum and a decreased concentration of glycogen in the hepatic tissues. Furthermore, the treatment of diabetic rats with CE, FP, and BP fractions of *V. amygdalina* leaves demonstrated a positive impact on glycemic response and glucose tolerance, as the levels of glucose and HbA1c in the serum were reduced while the hepatic glycogen level was significantly improved. The effects observed in this study were in accordance with previous studies, which have reported that *V. amygdalina* leaf extracts and fractions were able to produce anti-hyperglycemic responses in diabetic rats due to the presence of phytoconstituents, such as luteolin, vernodalol, vernomygdin, myrtenol, and others [45,46,47,48]. Several antidiabetic drugs work by limiting postprandial hyperglycemia via inhibition of α-amylase and α-glucosidase activity, preventing the breakdown of dietary carbohydrate to glucose [37,49]. Our results further showed that the CE, FP, and BP fractions of *V. amygdalina* were able to inhibit the activity of α-amylase and α-glucosidase in diabetic rats, providing further evidence of their anti-hyperglycemic effects as previously reported [50,51].

In vivo experimental evidence has shown that the diabetic state is characterized by insulin deficiency, resistance, and insensitivity [37,52]. In this study, the serum level of insulin was measured to investigate the effects of the three fractions of *V. amygdalina* on insulin secretion. In addition, the homeostasis model assessment of insulin resistance (HOMA-IR), homeostasis model assessment of β-cell function (HOMA-β), and quantitative insulin sensitivity check index (QUICKI) were calculated from the value of serum glucose and insulin. Similar to what has been previously reported [37,40,52], we observed that serum insulin, HOMA-β, and QUICKI were all decreased in diabetic rats, while HOMA-IR was increased. This was an indication that the STZ-induced diabetes model resulted in insulin deficiency and insensitivity in the rats. Treatments of DM rats with CE, FP, and BP fractions of *V. amygdalina* were able to reverse the observed reduction in serum insulin levels and calculated HOMA-β, similar to what was observed with the standard drug, metformin. The results were an indication that the fractions could improve insulin secretion and sensitivity, effects that may be due to the ability of the fractions to regenerate damaged β-cells or stimulate the release of insulin from the pancreas. Similar to our results, other studies have reported the ability of *V. amygdalina* extracts to improve insulin secretion and sensitivity [46,53]. Hepatic lipase is an enzyme that catalyzes the hydrolysis of triglycerides and phospholipids in lipoproteins, thus enhancing their clearance and metabolism [54]. Studies have shown that an increased hepatic lipase activity is strongly associated with insulin resistance and diabetes [55,56]. The findings of this study indicated an increase in the activity of hepatic lipase in STZ-induced diabetic rats compared with control. Treatment with the three fractions of *V. amygdalina* reversed the increase, with the bound phenolic fraction showing the most potent effect, further supporting the antidiabetic potential of the crude extracts and fractions of *V. amygdalina* leaves.

Hyperlipidemia characterized by hypercholesterolemia and hypertriglyceridemia are two of the most common lipid dysfunctions in the pathophysiology of diabetes and have been used as risk factors for cardiovascular disease [37,57]. Hypercholesterolemia in the diabetic state has been adduced to different mechanisms, including the inactivation of lipoprotein lipase [37,57,58], increased absorption of cholesterol from the intestine, increased biosynthesis of cholesterol, and increased fatty acid mobilization from adipose tissues [2,35,38]. Results have shown that the total cholesterol and LDL-cholesterol levels were significantly increased in the serum, while the HDL-cholesterol level was reduced in the STZ-induced rats compared with control, as previously reported in other studies [2,37,52]. Atherogenic risk assessment for cardiovascular disease is becoming vital for the management of lipid dysfunction linked with diabetes. Thus, the atherogenic coefficient (AC) and Castelli risk indexes I and II (CRI-I and CRI-II) are emerging as important risk predictors for cardiovascular disease. We further observed significant elevations in AC and CRI-I in untreated DM rats compared with the control. Only the crude extract and free phenolic fraction were able to reverse the changes observed in total cholesterol and HDL-cholesterol; furthermore, the free phenolic fraction was able to reduce the elevation in LDL-cholesterol in diabetic rats. These results further showed that all fractions of *V. amygdalina* were able to improve the atherogenic risk indices. The results described here point to the ability of *V. amygdalina* crude extract and fractions to reverse hyperlipidemia in the diabetic state, and thus, prevent atherosclerosis and other cardiovascular disease (CVD), as previously reported [45,48,59,60].

The liver is one of the organs that is adversely affected in the diabetic state, and evidence has shown that diabetes is associated with liver disorders. Diabetic hepatopathy may arise from several pathophysiologic mechanisms including, hyperglycemia, hyperlipidemia, and insulin deficiency and/or resistance, as well as compensatory hyperinsulinemia [61]. A very important feature of diabetic hepatopathy is the elevation in the activities of aminotransferases, such as AST, ALT, and alkaline phosphatase, in circulation [62,63]. Accordingly, in this study, we observed diabetes-induced hepatopathy characterized by elevations of AST, ALT, and ALP in the plasma, as well as histopathological impairments revealing vacuolar degeneration and disruption of portal areas. In addition, the three fractions of *V. amygdalina* were observed to reverse the STZ-induced elevations in AST, ALT, and ALP; however, only the crude extract and FP fraction were able to improve the histological alterations induced in the diabetic rats. This hepatoprotective effect of *V. amygdalina* has been previously reported [39,48,64,65], with evidence showing that it may be attributed to the ability of phytochemicals present in *V. amygdalina*, including luteolin, the vernoniosides, and myrtenol, to clear ROS and free radicals, thereby preventing oxidative damage to the liver [47,66].

It is a well-known fact that long-term hyperglycemia can mediate the over-production of ROS, which depletes cellular antioxidants, and subsequently results in oxidative stress and damage to macromolecules including lipids, proteins, and nucleic acids. The livers of diabetic animals in this study showed elevated levels of MDA (lipid peroxidation) and PCO (protein oxidation) with depleted levels of GSH, CAT, and SOD. Furthermore, treatment of diabetic rats with the three fractions of *V. amygdalina* induced a significant reduction in the levels of MDA and PCO while improving the activity of SOD, CAT, and GSH in a similar way to the effect seen with the standard drug, metformin. Previous studies have shown that *V. amygdalina* leaf extracts contain phytocompounds that are capable of scavenging free radicals, thus inhibiting lipid and protein oxidation and enhancing the antioxidant enzymes defense system in the livers of diabetic animals [17,46,67].

Oxidative stress and inflammation are known to co-exist and play an important role in the pathogenesis of diabetes and its complications. Hyperglycemia-induced ROS over-generation can activate increased formation of pro-inflammatory mediators, including c-reactive protein (CRP) and cytokines such as IL-1β and TNF-α, while the release of the anti-inflammatory cytokine, IL-10, was inhibited in the sera and livers of diabetic animals [37,68,69,70], as observed in this study. CRP is a commonly used biomarker for inflammation as it is synthesized in the hepatocytes in response to inflammation triggered by ROS generation in the diabetic state [71]. Glucose tolerance has been reported to be negatively impacted by pro-inflammatory mediators, with evidence showing that TNF-α can induce hepatic production of glucose while suppressing its uptake in the peripheral tissues [68]. IL-1β can elicit β-cell mass degradation [72] and compromises insulin sensitivity in hepatic cells [73]. Therefore, a strategy to reduce oxidative stress and inflammation may prevent β-cell loss, hyperglycemia, and glucose intolerance. Our results further demonstrated that treatment with the CE, FP, and BP fractions of *V. amygdalina* decreased the levels of CRP in the serum and TNF-α and IL-1β in the liver, while at the same time improving the hepatic level of IL-10. The anti-inflammatory effects seen with the *V. amygdalina* fractions are similar to the effect shown by the standard drug, metformin. This anti-inflammatory ability could be ascribed to the contribution of bioactive phytochemicals found in *V. amygdalina*. Luteolin [74], vernoniosides [75], vernodalin [76], vernomygdin, and vernodalol [66] have all been reported to exhibit strong anti-inflammatory effects in vitro and in vivo via mechanisms that involve the inactivation of NF-κB and the inhibition of pro-inflammatory cytokine production.

## 5. Limitations of the Study

It should be noted that the antidiabetic, antioxidant, anti-inflammatory, and hypolipidemic effects of the crude extract and the free phenol and bound phenol factions were evaluated using an STZ-induced type 1 rat model, which has been reported not to fully mimic the complex and chronic diabetes found in human. We acknowledge that this may be a major limitation of this study. However, it should be noted that the STZ-rat model is a good model to replicate most hyperglycemia-mediated micro- and macrovascular damage to organs that are seen in human diabetes, making it a very popular experimental model. Another limitation of this study was that it was conducted in male Wistar rats only. Therefore, our results may or may not be translatable to female Wistar rats. It has been reported that male rats and mice are more susceptible to STZ effects than their female counterparts [77,78]. In addition, other reports have indicated sex-related differences in the damage to the liver and the susceptibility to oxidative stress after various types of insults [79,80]. Thus, future studies must be cognizant of this, and both sexes of rats must be included.

## 6. Conclusions and Future Perspectives

The results obtained from this study indicated that CE, FP, and BP fractions of *V. amygdalina* leaves exhibited differential ameliorative effects in a type 1 diabetes mellitus model in rats. Specifically, our data revealed that the CE, FP, and BP fractions demonstrated antidiabetic effects by modulating glycemic response, reversing hyperlipidemia, attenuating hepatic injury, preventing oxidative stress, and inhibiting formation of proinflammatory mediators. Of the three fractions, the crude extracts demonstrated the most significant antidiabetic effect, similar or better than the effect seen with the standard drug, metformin, despite having a lower concentration of phytoconstituents compared with FP and BP fractions. This was not surprising, as evidence has shown that crude extracts of herbal plants often demonstrated greater efficacy in vitro and in vivo than isolated or refined fractions at similar doses due to the synergistic and holistic interactions of the complex phytoconstituents present in them [81]. This study was not only confirmatory of the antidiabetic effect of *V. amygdalina* crude extract but also has contributed new knowledge. Firstly, this was the first time that the antidiabetic, hypolipidemic, antioxidant, and anti-inflammatory effects of free and bound phenol fractions obtained from *V. amygdalina* have been reported. Secondly, the majority of the previous studies used much higher doses (200–500 mg/kg) to demonstrate efficacy; in this study, we use a much lower dose (50 mg/kg) to demonstrate efficacy and safety, as we did not observe any adverse effects in the rats. This became all the more important against the backdrop of toxicity and adverse effects that have been attributed to metformin. Despite overwhelming evidence of the efficacy of different *V. amygdalina* extracts in animal models of type 1 and type 2 diabetes, it was worrying that these results have not advanced to human studies. Only two studies [82,83] in the literature have reported the anti-hyperglycemic effect of *V. amygdalina* in healthy patients. Many reasons have been adduced for the existing gaps in the translation of animal model results to humans, including rigorous methodological and experimental design challenges, dose standardization for humans, a lack of evidence of safety for long-term use, as well as regulatory challenges. It is therefore imperative that robust, well-designed clinical trials must be conducted to confirm the efficacy and safety of *V. amygdalina* extracts in type 1 diabetic patients.

## Figures and Tables

**Table 1:** HPLC quantification of phenolic compounds in *Vernonia amygdalina*.

S/N	Phytochemicals	Crude Extract (mg/g)	Free Phenol (mg/g)	Bound Phenol (mg/g)
1	Benzophenone	ND	0.12 ± 0.01 ^a^	0.18 ± 0.01 ^a^
2	Vernomygdin	1.06 ± 0.01 ^b^	1.15 ± 0.01 ^a^	1.12 ± 0.03 ^a^
3	Vernodalin	0.51 ± 0.01 ^c^	0.60 ± 0.01 ^b^	0.67 ± 0.02 ^a^
4	Vernonioside	0.39 ± 0.01 ^b^	0.45 ± 0.01 ^a^	0.45 ± 0.01 ^a^
5	Garanal	0.34 ± 0.01 ^b^	0.37 ± 0.01 ^b^	0.44 ± 0.01 ^a^
6	Vernodalol	0.53 ± 0.02 ^b^	0.58 ± 0.03 ^b^	0.66 ± 0.02 ^a^
7	Myrtenol	0.39 ± 0.02 ^c^	0.56 ± 0.04 ^b^	0.78 ± 0.02 ^a^
8	Luteolin	1.41 ± 0.01 ^c^	1.83 ± 0.03 ^b^	2.31 ± 0.03 ^a^

Values are mean ± SEM (*n* = 3). Values in the same row with different superscripts are significantly different at *p* < 0.05. ND (not detected).

**Table 2:** Body weight changes, liver weight, and relative liver weight of rats across all experimental groups.

Treatment Groups	Initial Body Weight (g)	Final Body Weight (g)	Weight Change (%)	Liver Weight (g)	Relative Liver Weight (%)
Control	170.25 ± 9.25	209.50 ± 4.43	23.23 ± 4.63	8.60 ± 0.46	4.11 ± 0.29
DM	258.75 ± 10.01	227.00 ± 8.91	−12.24 ± 2.27	5.68 ± 0.64 ***	2.50 ± 0.24 ***
DM + CE	208.50 ± 3.32	226.50 ± 5.64	8.66 ± 2.76	7.60 ± 0.23 ^###, †^	3.36 ± 0.13 **^, ###, ††^
DM + FP	212.00 ± 4.97	225.00 ± 4.08	6.15 ± 1.11	7.67 ± 0.25 ^###^	3.41 ± 0.14 **^, ###, †^
DM + BP	194.00 ± 9.83	208.50 ± 5.07	7.58 ± 2.93	7.33 ± 0.36 ^##, ††^	3.52 ± 0.11 *^, ###^
DM + MET	204.75 ± 9.74	228.50 ± 9.54	11.69 ± 4.33	8.93 ± 0.24 ^###^	3.91 ± 0.20 ^###^
CE	181.75 ± 13.87	216.75 ± 14.66	19.38 ± 4.68	8.27 ± 0.82	3.81 ± 0.18
FP	198.05 ± 19.26	230.75 ± 16.28	16.49 ± 3.77	8.48 ± 0.74	3.67 ± 0.12
BP	178.50 ± 10.53	207 25 ± 9.32	16.19 ± 2.34	8.18 ± 0.74	3.94 ± 0.22

Data are expressed as mean ± SEM (*n* = 6). * *p* < 0.05, ** *p* < 0.01, and *** *p* < 0.001 as significant difference versus control. ^##^ *p* < 0.01 and ^###^ *p* < 0.001 as significant difference versus DM. ^†^ *p* < 0.05 and ^††^ *p* < 0.01 as significant difference versus DM + MET. BP (bound phenol fraction), CE (crude extract), DM (diabetes), FP (free phenol fraction), MET (metformin).

## Data Availability

The original contributions presented in this study are included in the article. Further inquiries can be directed to the corresponding author.

## Supplementary Materials

The following supporting information can be downloaded at: https://www.mdpi.com/article/10.3390/jox16020053/s1, Figure S1: HPLC chromatogram_Crude Extract; Figure S2: HPLC chromatogram_Free Phenol; Figure S3: HPLC chromatogram_Bound Phenol.

## Abbreviations

The following abbreviations are used in this manuscript:

AC	Atherogenic Coefficient
ALT	Alanine Aminotransferase
AST	Aspartate Aminotransferase
BP	Bound Phenol
CAT	Catalase
CE	Crude Extract
CRI-II	Castelli Risk Index II
CRI-I	Castelli Risk Index I
CRP	C-Reactive Protein
DM	Diabetes Mellitus
DTNB	5,5ʹ-dithiobis-2-nitrobenzoic Acid
FP	Free Phenol
GSH	Reduced Glutathione
HDL-C	High-Density Lipoprotein–Cholesterol
HOMA-IR	Homeostatic Model Assessment of Insulin Resistance
HOMA-β	Homeostatic Model Assessment of β-cell Function
IL	Interleukin
IκB	Inhibitor of Kappa B
IκKB	IκB Kinase
LDL-C	Low-Density Lipoprotein–cholesterol
MDA	Malondialdehyde
MET	Metformin
NF-κB	Nuclear Factor Kappa B
NO	Nitric Oxide
Nrf2	Nuclear Factor Erythroid 2-Related Factor 2
PCO	Protein Carbonyl
QUICKI	Quantitative Insulin Sensitivity Check Index
ROS	Reactive Oxygen Species
SOD	Superoxide Dismutase
STZ	Streptozotocin
T1DM	Type 1 Diabetes Mellitus
TG	Triglycerides
TNF-α	Tumor Necrosis Factor-α
