# Supplementary material for: Crude Extract and Phenol-Rich Fractions from Vernonia amygdalina Leaves Ameliorates Streptozotocin-Induced Type 1 Diabetes in Rats by Mitigating Hepatic Injury, Dyslipidemia, and Production of Oxido-Inflammatory Markers
Source: J Xenobiot. 2026 Mar 20;16(2):53. doi: 10.3390/jox16020053 (PMC13010673; doi:10.3390/jox16020053)
Supplement: Supplementary file 1 [file jox-16-00053-s001.zip › Figure S2.pdf]

Lab name: Bato Chemical Lab. Ltd
Client: Damilolola Phytochemicals
Client ID: Dami
Method: HPLC With UV
Description: CHANNEL 1
Column: uBondapak C18
Carrier: Acetonitrile / Water(70:30)
Data file: DAMILOLA VERNONIA AMYGDALINA, PHYTOCHEMICALS. ACTIVE TEST SAMPLE 2. RUN 2. 0106Y2021.CHR ()
Sample: Test Sample 2 , Run 2
Comments: 10g of Sample extracted with Acetonitrile , extract was stabilized with Ethyl Acetate , and made up to 25ml in standard flask with Acetonitrile . 5ul injected @ 2ml / min flow rate.

Temperature program:

Init temp Hold Ramp Final temp

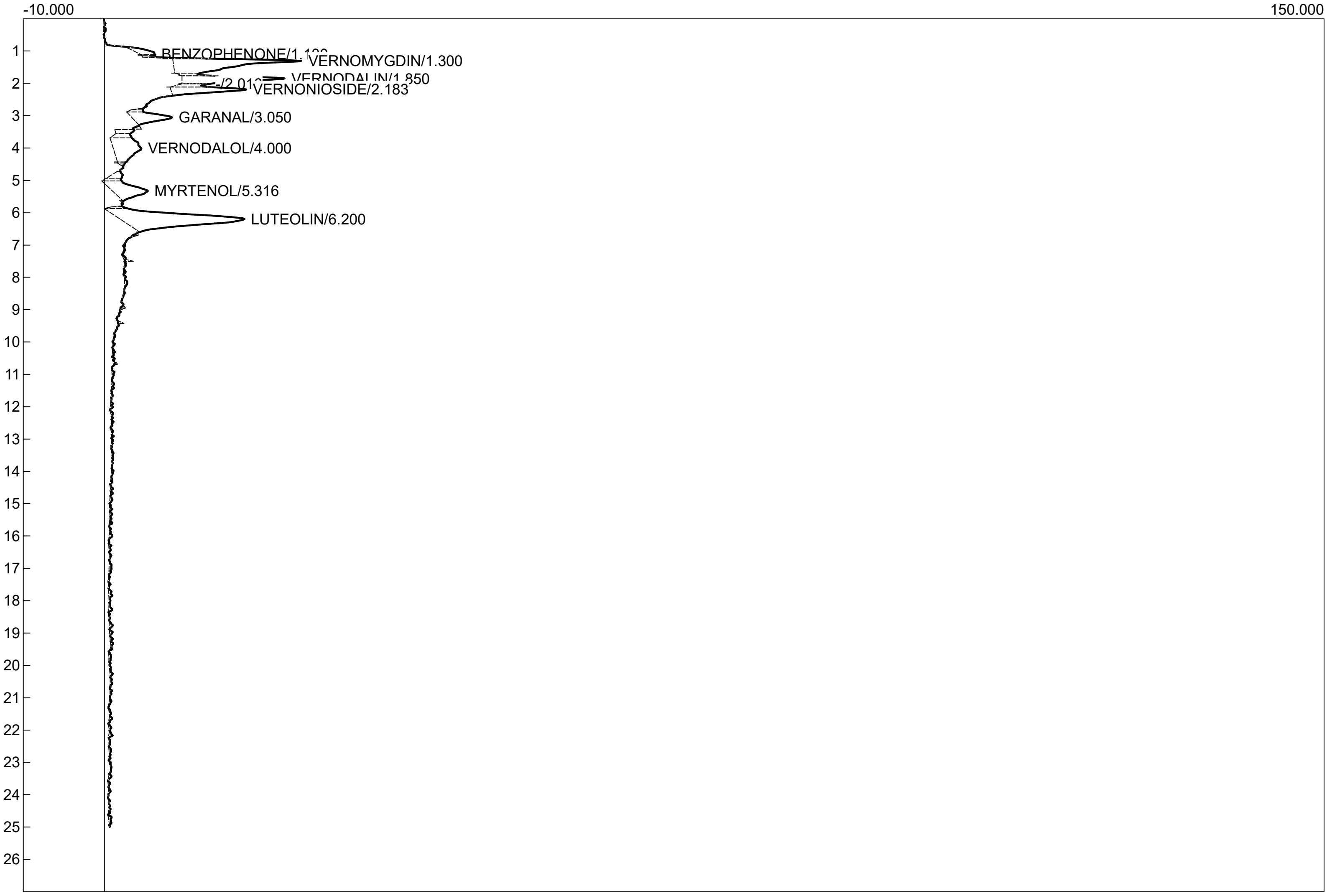

Component	Retention	Area	Height	External	Units
BENZOPHENONE	1.100	23.4495	1.787	0.0000	
VERNOMYGDIN	1.300	225.1830	15.839	0.0000	
VERNODALIN	1.850	120.1330	12.634	0.0000	
VERNONIOSIDE	2.183	87.6670	9.309	0.0000	
GARANAL	3.050	73.3370	4.983	0.0000	
VERNODALOL	4.000	117.1155	3.458	0.0000	
MYRTENOL	5.316	102.1280	4.385	0.0000	
LUTEOLIN	6.200	352.8310	15.265	0.0000	
		1101.8440		0.0000	
